# Comprehensive assessment of the safety of olodaterol 5 µg in the Respimat^®^ device for maintenance treatment of COPD: comparison with the long-acting β_2_-agonist formoterol

**DOI:** 10.1038/s41533-017-0059-1

**Published:** 2017-10-23

**Authors:** Andrea Koch, Henrik Watz, M. Reza Maleki-Yazdi, Ulrich Bothner, Kay Tetzlaff, Florian Voß, Lorcan McGarvey

**Affiliations:** 1 Medizinische Klinik und Poliklinik V, Klinikum der Ludwig-Maximilians-Universität, Munich, Germany; 2 German Center for Lung Research (DZL), Klinikum der Ludwig-Maximilians-Universität, Munich, Germany; 3 Pulmonary Research Institute at Lung Clinic Grosshansdorf, Airway Research Center North, German Center for Lung Research (DZL), Grosshansdorf, Germany; 4 Division of Respiratory Medicine, Women’s College Hospital, University of Toronto, Toronto, ON Canada; 5 Boehringer Ingelheim Pharma GmbH & Co. KG, Ingelheim, Germany; 6 Centre for Infection and Immunity, School of Medicine, Dentistry and Biomedical Sciences, Queen’s University Belfast, Belfast, UK

## Abstract

This analysis provides a comprehensive clinical assessment of the long-term safety of the licensed dose of olodaterol (5 µg once daily [QD] via Respimat^®^ inhaler) in patients with chronic obstructive pulmonary disease by exploring the occurrence of acknowledged side effects of long-acting β_2_-agonists as well as those included in the olodaterol and formoterol labels. We analysed pooled data from two replicate, double-blind studies of olodaterol (5 µg QD via Respimat^®^) compared to formoterol (12 µg twice daily [BID]) or placebo over 48 weeks (1222.13, NCT00793624; 1222.14, NCT00796653). Patients could continue their background treatment. The analysis considered adverse events (AEs) typically associated with β_2_-agonists, including cardiovascular events, as well as administration-related events. Descriptive statistics were provided for the incidence of AEs and aggregated AEs. The analysis included 1379 patients: 460 placebo, 459 olodaterol and 460 formoterol; AEs were reported by 70.9, 71.7 and 69.1% of patients, respectively. Exposure-adjusted incidence rates of cardiac AEs (arrhythmia and myocardial ischaemia) and cough were numerically lower in the olodaterol group than the formoterol group, while nasopharyngitis, throat irritation, metabolism and psychiatric disorders were numerically higher in the olodaterol group. The most frequent event in the olodaterol group was nasopharyngitis (placebo 8.0%; olodaterol 12.9%; formoterol 10.0%). Except for cough (incidence rate ratio of 0.46 [95% confidence interval 0.24, 0.89] in favour of olodaterol), there were no significant differences between active groups. In conclusion, olodaterol 5 µg QD was well tolerated over 48 weeks with a typical β_2_-agonist safety profile comparable to formoterol 12 µg BID.

## Chronic lung disease: Drug safety confirmed

The long-term safety of a recently-certified inhaled drug for the treatment of chronic lung disease is verified by scientists in Germany. The management of chronic obstructive pulmonary disease involves inhaled drugs called long-acting β_2_ agonists (LABAs). The most recently-certified LABA is called olodaterol. Andrea Koch at the Klinikum der Ludwig-Maximilians-Universität in Munich, Germany, and co-workers have clarified the long-term safety of olodaterol during two trials that compared it with formoterol, an existing LABA. The trials lasted 48 weeks and involved 1379 patients—460 were given a placebo, 459 received 5 µg olodaterol via a Respimat device, and 460 received formoterol. Side effects from olodaterol were no more severe or persistent than those associated with formoterol. Reassuringly, the new drug also displayed similar or slightly lower incidence of cardiovascular side effects such as heart palpitations compared with formoterol.

## Introduction

Bronchodilators, including long-acting β_2_-agonists (LABAs), are central to the symptom management of chronic obstructive pulmonary disease (COPD).^1^ The twice-daily (BID) inhaled LABAs formoterol and salmeterol have been available for the treatment of COPD since the late 1990s and effectively improve lung function, symptoms of breathlessness and quality of life.^2^ Of the two agents, formoterol (12 µg BID) also has the benefit of a faster onset of action.^3^ Owing to the extensive experience with these established agents, they have well-defined safety and tolerability profiles that have been used as reference for β_2_-agonist class effects when evaluating other LABAs.^4,5^

Inhaled LABAs are generally well tolerated but have acknowledged class side effects, including palpitations, headache, nasopharyngitis and tremor.^1,6^ There are also potential cardiovascular risks such as arrhythmia and myocardial ischaemia associated with LABAs, particularly in patients with concomitant cardiac disorders, which are common co-morbidities in patients with COPD.^7^

Olodaterol is a novel once-daily (QD) LABA that has recently been approved and is available to clinicians via the Respimat^®^ device (Boehringer Ingelheim GmbH & Co. KG, Ingelheim, Germany) (5 µg QD).^8^ Given that olodaterol has only been available since mid-2014, post-marketing experience is currently limited; however, long-term safety data for olodaterol (5 and 10 µg QD) are available from a large clinical trial programme.^8,9^ We conducted an analysis to specifically assess the safety profile of the approved and marketed dose of olodaterol (5 µg QD) in comparison to formoterol (12 µg BID). This analysis goes beyond a standard safety review by considering specific adverse events (AEs) associated with the class, as well as those included in the label for formoterol and olodaterol, to provide clinicians with a clear understanding of its safety profile and place olodaterol into context with other LABAs. The pooled safety analysis was pre-specified but without incidence rates, and additional grouped terms were added. The higher dose of olodaterol in these studies (10 µg) has not been discussed in this paper.

## Results

A total of 1379 patients were included in this analysis: 460 randomised to placebo, 459 to olodaterol 5 µg and 460 to formoterol 12 µg. In general, the treatment groups were well balanced with respect to demographic and baseline patient characteristics (Table 1), although the placebo group had a slightly larger proportion of patients with a COPD severity of Global Initiative for Chronic Obstructive Lung Disease (GOLD) 4 (10.0 versus 6.8% in the olodaterol group and 8.3% in the formoterol group). A substantial proportion of patients in each group had cardiac (18.9, 19.0 and 20.0%, respectively) or vascular (42.0, 41.2 and 43.7%, respectively) co-morbidities, reflecting the severity of COPD in this study patient population.

**Table 1:** Demographic and baseline patient characteristics (treated population)

Characteristics	Placebo (*n* = 460)	Olodaterol 5 µg (*n* = 459)	Formoterol 12 µg (*n* = 460)
Male, *n* (%)	375 (81.5)	364 (79.3)	371 (80.7)
Mean (SD) age, years	63.9 (8.1)	63.7 (8.9)	64.9 (8.4)
Smoking status, *n* (%)			
Ex-smoker	301 (65.4)	303 (66.0)	305 (66.3)
Current smoker	159 (34.6)	156 (34.0)	155 (33.7)
Mean (SD) smoking history, pack-years	43.7 (25.0)	43.0 (25.0)	45.1 (26.0)
Mean (SD) BMI (kg/m^2^)	25.2 (5.2)	25.3 (5.7)	24.9 (5.2)
Co-morbidities, *n* (%)			
Cardiac	87 (18.9)	87 (19.0)	92 (20.0)
Vascular	193 (42.0)	189 (41.2)	201 (43.7)
GOLD, *n* (%)			
1 (≥30%)	1 (0.2)	2 (0.4)	3 (0.7)
2 (50–<80%)	242 (52.6)	255 (55.6)	247 (53.7)
3 (30–<50%)	171 (37.2)	171 (37.3)	172 (37.4)
4 (<30%)	46 (10.0)	31 (6.8)	38 (8.3)
Baseline pulmonary medication, *n* (%)			
Any pulmonary medication	377 (82.0)	391 (85.2)	385 (83.7)
SAMA	136 (29.6)	144 (31.4)	133 (28.9)
LAMA	118 (25.7)	117 (25.5)	117 (25.4)
SABA	217 (47.2)	218 (47.5)	220 (47.8)
LABA	170 (37.0)	168 (36.6)	173 (37.6)
Oral β-adrenergics	4 (0.9)	6 (1.3)	2 (0.4)
Leukotriene receptor antagonists	11 (2.4)	9 (2.0)	2 (0.4)
Mucolytics	31 (6.7)	31 (6.8)	34 (7.4)
Oxygen	4 (0.9)	0 (0.0)	1 (0.2)
ICS	227 (49.3)	222 (48.4)	210 (45.7)
Oral steroids	7 (1.5)	12 (2.6)	5 (1.1)
Xanthines	78 (17.0)	88 (19.2)	80 (17.4)

*SD* standard deviation, *BMI* body mass index, *GOLD* Global Initiative for Chronic Obstructive Lung Disease, *SAMA* short-acting muscarinic antagonist, *LAMA* long-acting muscarinic antagonist, *SABA* short-acting β-agonist, *LABA* long-acting β_2_-agonist, *ICS* inhaled corticosteroid

**Fig. 1:**
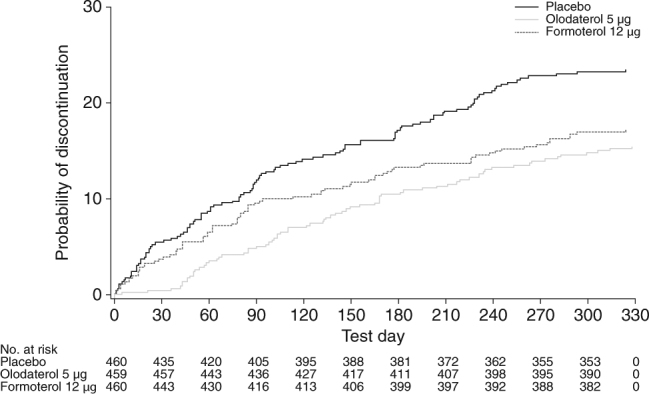
Probability of discontinuation with placebo, olodaterol and formoterol. Cox regression analysis shows a significant difference from placebo for olodaterol 5 μg (*P* = 0.0013) and for formoterol treatment (*P* = 0.0180)

A greater proportion of patients discontinued treatment in the placebo group (23.5%) than the olodaterol 5 µg (15.9%) or formoterol 12 µg (18.0%) groups. A Kaplan–Meier analysis of time to discontinuation for any reason showed that the probability of discontinuation was significantly higher with placebo than olodaterol (*P* = 0.0013) and formoterol (*P* = 0.0180) throughout the study (Fig. 1). Discontinuations were similar between olodaterol and formoterol, with the Kaplan–Meier curve for time to discontinuation for olodaterol slightly below the one for formoterol over the whole observational period.

### Summary of safety outcomes

**Fig. 2:**
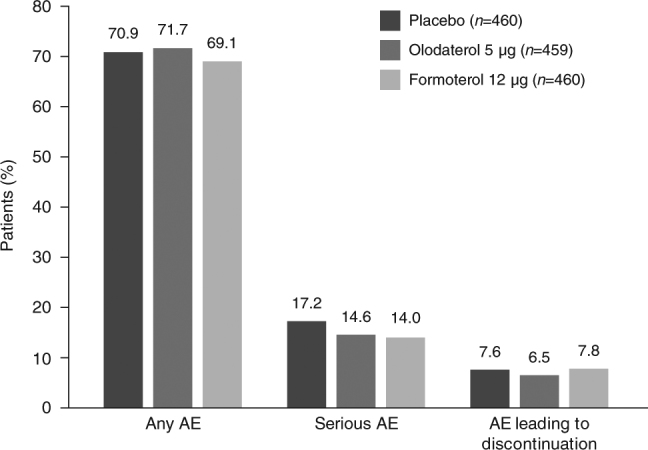
Overall incidence of adverse events (AEs)

Patients with any AE were reported at 70.9, 71.7 and 69.1% in the placebo, olodaterol and formoterol groups, respectively. The incidence of serious AEs was slightly lower in the olodaterol and formoterol groups than with placebo. Ten patients in each group (2.2%) experienced fatal AEs. The incidence of AEs leading to discontinuation was lowest in the olodaterol 5 µg group (Fig. 2). The incidence of investigator-defined drug-related AEs was lower in the olodaterol 5 µg group (6.1%) than the placebo and formoterol 12 µg groups (9.1 and 11.1%, respectively).

### Cardiovascular AEs

The crude incidences of arrhythmia and myocardial ischaemia events were lower in the olodaterol 5 µg group than the placebo and formoterol 12 µg groups (Table 2). Crude incidence of the combined torsade de pointes/QT prolongation Standardised MedDRA Query (SMQ) was five patients (1.1%) in each group. All cases were QT prolongation reported as an AE in any of the frequently repeated electrocardiogram study measurements; there was no occurrence of Torsades de pointes arrhythmia in any of the treatment groups.

**Table 2:** Incidence of cardiovascular, general and administration-related AEs (formoterol-labelled β_2_-agonist class side effects)

AE, *n* (%)	Placebo	Olodaterol 5 µg	Formoterol 12 µg
	(*n* = 460)	(*n* = 459)	(*n* = 460)
Cardiovascular			
Tachyarrhythmias (including extrasystoles)	16 (3.5)	10 (2.2)	15 (3.3)
Supraventricular (including atrial fibrillation)	12 (2.6)	5 (1.1)	10 (2.2)
Ventricular	6 (1.3)	5 (1.1)	9 (2.0)
Torsade de pointes/QT prolongation	5 (1.1)	5 (1.1)	5 (1.1)
Ischaemic heart disease	12 (2.6)	4 (0.9)	7 (1.5)
Myocardial infarction	3 (0.7)	1 (0.2)	4 (0.9)
Other ischaemic heart disease (non-infarction)	9 (2.0)	3 (0.7)	3 (0.7)
Hypotension	1 (0.2)	0 (0.0)	2 (0.4)
Hypertension	16 (3.5)	14 (3.1)	10 (2.2)
Palpitations	8 (1.7)	4 (0.9)	10 (2.2)
Peripheral oedema	6 (1.3)	8 (1.7)	5 (1.1)
Respiratory and administration related			
Cough	23 (5.0)	13 (2.8)	27 (5.9)
Bronchospasm	1 (0.2)	0 (0.0)	2 (0.4)
Nasopharyngitis	37 (8.0)	59 (12.9)	46 (10.0)
Throat and other application-site irritation	52 (11.3)	69 (15.0)	58 (12.6)
Gastrointestinal			
Nausea	7 (1.5)	4 (0.9)	3 (0.7)
Dry mouth	6 (1.3)	7 (1.5)	5 (1.1)
Metabolism			
Hypokalaemia	1 (0.2)	0 (0.0)	0 (0.0)
Hyperglycaemia/new-onset diabetes mellitus	6 (1.3)	7 (1.5)	4 (0.9)
Musculoskeletal			
Arthralgia/myalgia/muscle weakness	46 (10.0)	50 (10.9)	52 (11.3)
Muscle spasm	6 (1.3)	8 (1.7)	10 (2.2)
Nervous system			
Dizziness	11 (2.4)	10 (2.2)	10 (2.2)
Tremor	1 (0.2)	2 (0.4)	3 (0.7)
Headache	18 (3.9)	16 (3.5)	16 (3.5)
Psychiatric			
Nervousness	1 (0.2)	0 (0.0)	0 (0.0)
Restlessness/agitation	1 (0.2)	0 (0.0)	1 (0.2)
Anxiety	3 (0.7)	3 (0.7)	1 (0.2)
Insomnia	1 (0.2)	5 (1.1)	3 (0.7)

* AE* adverse event

**Fig. 3:**
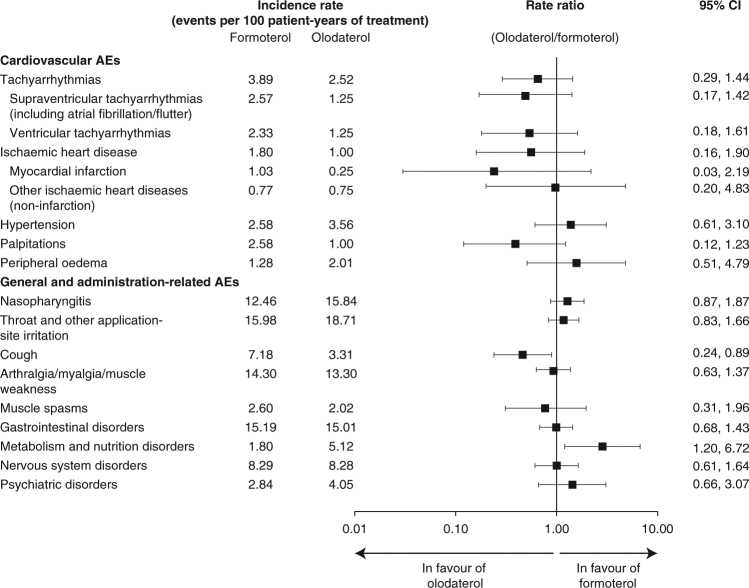
Forest plot showing the exposure-adjusted rate and ratio for adverse events (AEs) of interest comparing olodaterol 5 µg with formoterol 12 µg. *CI* confidence interval

The majority of exposure-adjusted incidence rate ratios (RRs) for the cardiovascular AE groups (including tachyarrhythmia and ischaemic heart disease) were lower with olodaterol compared to formoterol. However, differences were not statistically significant since the 95% confidence intervals (CIs) included 1.0 (Fig. 3).

#### General AEs and those associated with drug administration

Overall, the crude incidences of other AE groups and those associated with drug administration were similar across the three treatment arms (Table 2). The crude incidences of nasopharyngitis and throat and other application-site irritation were higher in the olodaterol 5 µg group (12.9 and 15.0%, respectively) than placebo (8.0 and 11.3%, respectively) and the formoterol 12 µg group (10.0 and 12.6%, respectively). The crude incidence of cough was lower with olodaterol (2.8%) than placebo (5.0%) and formoterol (5.9%).

The extent of exposure-adjusted RRs was in line with the observed crude incidence differences comparing olodaterol 5 µg to formoterol 12 µg. Olodaterol 5 µg was associated with numerically higher RRs of nasopharyngitis, throat and other application-site irritation, metabolism and psychiatric disorders (Fig. 3). Formoterol was associated with numerically higher RRs for cough, musculoskeletal, gastrointestinal and nervous system disorders. However, with the exception of cough (single MedDRA Preferred Term) and metabolism and nutrition disorders (collection of AEs in MedDRA System Organ Class), the 95% CIs for RRs of AEs between olodaterol and formoterol included 1.0 (i.e., were not statistically significant). There were low incidence rates for metabolic and nutrition disorders System Organ Class (mainly hypoglycaemia) with no substantial difference in the crude incidence for olodaterol or formoterol compared to placebo (Table 2).

#### Rescue medication

Throughout the 48-week study period, patients receiving olodaterol 5 µg or formoterol 12 µg took significantly less daytime and night-time rescue medication than those receiving placebo (*P* < 0.05). At Week 48, the weekly mean (standard error [SE]) number of puffs during the daytime was 1.322 (0.068), 1.053 (0.069) and 1.062 (0.069) with placebo, olodaterol 5 µg and formoterol 12 µg, respectively. The common baseline mean (SE) was 1.221 (0.039). For night-time rescue medication use, the weekly mean (SE) number of puffs was 2.023 (0.093), 1.492 (0.093) and 1.582 (0.093) with placebo, olodaterol 5 µg and formoterol 12 µg, respectively. The common baseline mean (SE) was 1.943 (0.053).

There were no relevant differences between the active treatment arms in rescue medication usage.

## Discussion

### Main findings

This analysis of pooled data from two replicate, 48-week, phase III studies comparing olodaterol 5 µg QD with formoterol 12 µg BID or placebo in patients with moderate to very severe COPD (GOLD 2–4) showed that the overall incidence and types of most frequent AEs were similar in the olodaterol, placebo and formoterol groups. Nasopharyngitis, which is a known side effect of Striverdi^®^ Respimat^®^, occurred more frequently with olodaterol 5 µg than placebo.^8^ Importantly, cardiovascular events occurred infrequently and generally with a similar or lower incidence with olodaterol 5 µg than placebo or formoterol 12 µg. This provides reassurance of the cardiovascular safety of olodaterol among the β_2_-agonist class in COPD treatment.

### Interpretation of findings in relation to previously published work

Our analysis considered AEs typically associated with the inhaled β_2_-agonist class. The data originate from randomised controlled studies in which study medication was added on to existing background treatment across all groups, allowing the effect of the maintenance treatment LABA to be tested on top of a realistic COPD background therapy. The fact that there was no substantial difference with olodaterol and placebo in the incidence of AEs supports the acceptable general safety profile of this medication. The lack of any substantial difference between active treatments and placebo may have been influenced, however, by rescue medication usage; throughout the course of the study, the use of rescue medication (salbutamol) was higher in the placebo group than the active treatment arms, increasing patient exposure to the inherent β_2_-agonist risks.

The direct comparison of exposure-adjusted AE incidences between the olodaterol and formoterol groups indicated that both drugs have a similar safety profile overall. The safety profile of olodaterol in this analysis also appears similar to that reported for the LABAs indacaterol^5,10^ and vilanterol^11^ in patients with COPD. Donohue et al. reported that the most common AEs associated with indacaterol were worsening of COPD, nasopharyngitis and headache, and that there was no increase in the incidence of major adverse cardiovascular events with indacaterol versus placebo (0.69–1.10% versus 1.44%).^10^ In an analysis of the cardiovascular and cerebrovascular safety profiles in patients with COPD, indacaterol was similar to formoterol, salmeterol, tiotropium and placebo.^5^

### Strengths and limitations of this study

Co-morbidities are common in patients with COPD, including cardiac, metabolic, psychiatric, neurological and musculoskeletal disorders,^12^ and this was indeed the case in our study. Therefore, it is difficult to establish a causal drug relationship to specific AEs when the population is at high risk of such events merely from their pre-existing diseases. Consequently, this analysis included all reported AEs irrespective of the study investigators’ assessment of causality. For information, the incidence of drug-related AEs as detailed by the study investigators was low and not substantially different for olodaterol, placebo and formoterol (6.1, 9.1 and 11.1%, respectively).

Patient selection and treatment exposure in these studies were close to real-world data. Given the fact that large, long-term, blinded, randomised controlled trials are extremely difficult and expensive to conduct in COPD, a 1-year observation may not be long enough to detect very rare AEs. This is, however, usually the domain of post-marketing pharmacovigilance and not in scope of an everyday safety profile comparison. Overall, the nature and frequency of events reported in our studies were consistent with the disease under study and thus confirmed that olodaterol 5 µg QD has an acceptable safety and tolerability profile in patients with moderate to severe COPD, similar to that of the well-established LABA formoterol 12 µg BID.

### Implications for future research, policy and practice

These safety profiling data add support to the previously published data with olodaterol.^9^ However, as olodaterol is relatively new to the market (as with other once-daily LABAs), continued assessment will be required to ensure that its safety profile remains clearly defined.

## Conclusions

Olodaterol 5 µg QD via Respimat^®^ was well tolerated over 48 weeks, with a typical β_2_-agonist safety profile comparable to formoterol 12 µg BID dry powder inhalation. The tolerability is also supported by the lowest discontinuation rate for olodaterol among all treatments. Based on blinded, randomised, parallel-group, placebo-controlled and formoterol-controlled, long-term clinical data, the incidence of AEs was similar with olodaterol 5 µg QD for serious AEs and cardiovascular AEs compared with formoterol 12 µg BID. Mortality was low and similar across all treatment groups (approximately 2.2%, which is a usual mortality rate over 1 year in a COPD population). These data give reassuring information about the safety of olodaterol as a LABA option in moderate to severe COPD maintenance treatment. The data from this analysis suggest that olodaterol is an adequate alternative to formoterol for COPD maintenance treatment.

## Methods

This analysis included pooled data from two replicate, multicentre, double-blind, phase III studies of olodaterol (5 µg QD and 10 µg QD via Respimat^®^) compared to formoterol (12 µg BID dry powder inhalation) or placebo over 48 weeks (1222.13, NCT00793624; 1222.14, NCT00796653). Both devices (the olodaterol Respimat^®^ and the formoterol dry powder inhaler) were used together with the respective placebo device (double-dummy administration). The blinded formoterol study medication contained Foradil^®^.^13^ The studies included patients with moderate to very severe COPD (GOLD 2–4) who were aged ≥40 years and were current or ex-smokers (with a smoking history >10 pack-years). Patients with a history of asthma were excluded. With the exception of LABAs, patients continued with their existing COPD maintenance treatment, including short-acting muscarinic antagonists, long-acting muscarinic antagonists, corticosteroids and xanthines. All patients were provided with salbutamol as rescue medication. Full details of the study methodology have been published elsewhere.^14^ In total across the two studies, 1838 patients were randomised to treatment (225 to placebo, 227 to olodaterol 5 µg, 225 to olodaterol 10 µg and 227 to formoterol 12 µg in study 1222.13; 235 to placebo, 232 to olodaterol 5 µg, 234 to olodaterol 10 µg and 233 to formoterol 12 µg in study 1222.14). This analysis focuses on the licensed dose of olodaterol (5 µg QD via Respimat^®^).

After a baseline visit, patients attended study visits following 2, 6, 12, 18, 24, 32, 40 and 48 weeks of treatment. AEs were recorded throughout the study, irrespective of investigator-reported causality.

### Ethics

The studies were performed in accordance with the Declaration of Helsinki, International Conference on Harmonisation Harmonised Tripartite Guideline for Good Clinical Practice and local regulations. The protocols were approved by the authorities and ethics committees of the respective institutions, and signed informed consent was obtained from all patients.

### Selection of outcomes

Outcomes for comparing olodaterol with formoterol in this analysis were selected based on the typical β_2_-agonist class side effects, as exemplified by the formoterol (Foradil^®^) label.^13^ Side effects in the olodaterol label were also included in the analysis; however, given the much shorter time that olodaterol has been available, the range of events is less extensive than for formoterol.

The list of labelled side effects in the formoterol Summary of Product Characteristics was used as a basis to determine safety search terms, as shown in Table 3. Although not included in the formoterol label, nasopharyngitis was also evaluated in this analysis, as it was reported with olodaterol at a higher frequency than placebo in the clinical programme. While myocardial infarction is not included in the label for either formoterol or olodaterol, it was considered an important event for inclusion in this analysis. The formoterol-labelled events of bronchospasm paradoxical and taste disorders (dysgeusia) do not appear in the results as there were no occurrences in the study database in any treatment group. Events relating to hypersensitivity were not regarded typical to β_2_-agonist pharmacology but rather substance specific and, therefore, are not considered as part of the scope of this article.

**Table 3:** Label-recognised β_2_-agonist-related side-effect terms for formoterol and olodaterol^8,13^ and database event search terms

Organ/body system	Foradil^®^ (formoterol) SPC side effects	Striverdi^®^ Respimat^®^ (olodaterol) SPC side effects	Study database event search terms (groups)
Cardiovascular	Tachycardia, cardiac arrhythmias e.g., atrial fibrillation, supraventricular tachycardia, extrasystoles	–	Tachyarrhythmias (including supraventricular and ventricular tachyarrhythmias) (SMQ)
			• Supraventricular tachyarrhythmias (SMQ)
			• Ventricular tachyarrhythmias (SMQ)
	Palpitations	–	Palpitations (PV)
	Angina pectoris	–	Ischaemic heart disease (SMQ)
			• Myocardial infarction (SMQ)
			• Other ischaemic heart disease (SMQ)
	Prolongation of QTc interval	–	Torsade de pointes/QT prolongation (SMQ)
	Increased blood pressure (including hypertension)	Hypertension	Hypertension (SMQ)
	Variations in blood pressure	–	Hypotension (PV)
	Peripheral oedema	–	Peripheral oedema (PV)
Respiratory and administration related	Bronchospasm, acute asthma exacerbation^a^	–	Bronchospasm (broad) (PV)
	Paradoxical bronchospasm	–	Bronchospasm paradoxical (PT)
	Throat irritation	–	Throat and other application site irritation (PV)
	Cough	–	Cough (PT)
	–	Nasopharyngitis	Nasopharyngitis (PT)
Gastrointestinal	Dry mouth	–	Dry mouth (PV)
	Nausea	–	Nausea (PV)
Metabolic	Hypokalaemia	–	Hypokalaemia (PV)
	Hyperglycaemia	–	Hyperglycaemia/new–onset diabetes mellitus (SMQ)
Musculoskeletal	Myalgia	Arthralgia	Arthralgia/myalgia/muscle weakness (PV)
	Muscle cramp	–	Muscle spasm (PV)
Nervous system	Headache	–	Headache (PV)
	Tremor	–	Tremor (PV)
	Dizziness	Dizziness	Dizziness (PV)
	Dysgeusia	–	Taste disorders (PV)
Psychiatric	Agitation	–	Restlessness/agitation (PV)
	Anxiety	–	Anxiety (PV)
	Nervousness	–	Nervousness (PV)
	Restlessness	–	Restlessness/agitation (PV)
	Insomnia	–	Insomnia (PV)

*MedDRA* Medical Dictionary for Regulatory Activities, *PT* MedDRA preferred term, *PV* sponsor-defined pharmacovigilance end point, *SMQ* standardised MedDRA query

^a^ Not specific to COPD indication, relates to bronchospasm (note: formoterol has an indication and label for both asthma and COPD)

### Statistical methodology

Descriptive statistics were provided for the incidence of AEs and aggregated AE groups (based on Medical Dictionary for Regulatory Activities SMQs, or sponsor-defined pharmacovigilance end points where SMQs were not available for the medical AE concept of interest). AE incidences are described with their crude incidence (percentage of patients with the event out of all patients in the treatment group) and exposure-adjusted incidence rate (number of events per 100 patient-years at risk with the drug) in order to adjust for different exposure between the treatment arms in case of differential study discontinuation. Exposure-adjusted RRs and their 95% CIs are calculated based on a Cochran–Mantel–Haenszel method stratified by study to compare olodaterol versus formoterol treatment groups. AEs of interest are displayed with their RR and 95% CI in a forest plot. Time to early discontinuation of patients from the study was compared with a Cox regression analysis of treatment effect using baseline long-acting muscarinic antagonist concomitant medication (tiotropium) use as a stratification factor. This is a post hoc analysis and no formal statistical testing of hypotheses was performed. The assessment of statistical significance via 95% CIs of the RR is, therefore, considered exploratory and no adjustment for multiple testing was done.

### Data availability

Researchers can use the following link: http://trials.boehringer-ingelheim.com to find information in order to request access to the clinical study data, for this and other listed studies, after the submission of a research proposal and according to the terms outlined in the website.
